# Comparative Efficacy and Safety of Vasopressors and Inotropes in Acute Myocardial Infarction-Related Cardiogenic Shock: A Systematic Review and Network Meta Analysis of Randomized and Observational Studies

**DOI:** 10.7759/cureus.102204

**Published:** 2026-01-24

**Authors:** Ahmed Osman Hassan Ali, Farrukh Ameer, Ahmad A Ibrahim, Mohammad S Ali, Tamer A Abdelhamid, Mohamed Hany Elmasry, Fahd Alrumaih, Khaled Ahmed Reda Soliman, Ali Alghannami, Sara S Abdalla, Alaa A Hassan

**Affiliations:** 1 Critical Care, Dr. Soliman Fakeeh Hospital, Riyadh, SAU; 2 Adult Cardiology, Dr. Soliman Fakeeh Hospital, Riyadh, SAU; 3 Emergency Medicine, Riyadh Hospital, Riyadh, SAU; 4 Critical Care, Ministry of Health-Imam Abdulrahman Alfaisal Hospital, Riyadh, SAU; 5 Intensive Care Unit and Anesthesiology, Care Medical Hospital, Riyadh, SAU; 6 Surgery, Prince Sattam Bin Abdulaziz University, Riyadh, SAU; 7 Emergency Medicine, Armed Forces Hospital Southern Region, Khamis Mushait, SAU; 8 Internal Medicine, Ministry of Health, Riyadh, SAU; 9 General Medicine, Northampton General Hospital, Northampton, GBR; 10 Emergency Medicine, University Hospital Kerry, Tralee, IRL

**Keywords:** acute myocardial infarction, cardiogenic shock, dobutamine, inotropes, milrinone, mortality, network meta-analysis, norepinephrine, vasopressors

## Abstract

This systematic review and network meta-analysis evaluated the comparative efficacy and safety of vasopressors and inotropes in patients with cardiogenic shock complicating acute myocardial infarction. Electronic databases were searched from inception to December 2025 for randomized controlled trials (RCTs) and observational studies that compared norepinephrine, epinephrine, dopamine, dobutamine, milrinone, and levosimendan. The primary outcome was all-cause mortality, and the secondary outcomes included arrhythmia and refractory shock. Data were synthesized using a frequentist random-effects network meta-analysis. The certainty of evidence was assessed using the GRADE framework. In total, 14 studies (N = 5,157) were included, comprising six RCTs and eight observational studies. The network geometry was connected via bridging observational data. In the modern era (post-2000), no single agent significantly reduced mortality compared with others. Milrinone and dobutamine were equivalent in terms of in-hospital mortality (odds ratio (OR) = 0.90, 95% confidence interval (CI) 0.69-1.19; high certainty). Norepinephrine was associated with a lower arrhythmic risk than dopamine and was superior to epinephrine, which significantly increased the risk of refractory shock (OR = 8.24, 95% CI = 1.61-42.18; moderate certainty) and mortality (OR = 1.63 vs. norepinephrine). Historical studies have shown large effect sizes that have diminished over time (the Proteus phenomenon). Norepinephrine is the preferred vasopressor due to its safety profile; however, the choice of inotrope between milrinone and dobutamine should be guided by patient physiology rather than survival expectations. Future research requires large-scale trials to detect modest mortality benefits in patients with diabetes.

## Introduction and background

Cardiogenic shock (CS) is a critical tissue hypoperfusion resulting from severe cardiac dysfunction, most commonly secondary to acute myocardial infarction (AMI) [1]. Despite advancements in early reperfusion strategies and mechanical circulatory support (MCS), in-hospital mortality rates remain prohibitively high, ranging from 40% to 50% [2]. Hemodynamic collapse in CS initiates a vicious cycle of hypotension, systemic inflammation, and multiorgan failure, requiring rapid pharmacological stabilization to maintain perfusion pressure and cardiac output [3].

Vasoactive agents, including vasopressors and inotropes, are the cornerstone of medical management in CS [4]. Vasopressors, such as norepinephrine, epinephrine, and dopamine, increase systemic vascular resistance to restore mean arterial pressure (MAP), whereas inotropes, such as dobutamine, milrinone, and levosimendan, enhance myocardial contractility to augment cardiac output [5]. Current clinical guidelines, including those from the European Society of Cardiology and the American Heart Association, recommend norepinephrine as the first-line vasopressor [1,6]. This recommendation is driven by evidence from the SOAP II trial, which demonstrated a lower incidence of arrhythmias with norepinephrine than with dopamine, although no significant difference in overall mortality was observed [7].

However, the optimal choice of inotropic support is contentious, as dobutamine, a synthetic catecholamine, is used as the first-line inotrope due to its balanced effects on contractility and vasodilation [6]. Milrinone, a phosphodiesterase-3 inhibitor, offers an alternative mechanism independent of beta-adrenergic receptors, potentially advantageous in patients with chronic beta-blocker therapy or pulmonary hypertension, but is limited by a longer half-life and risk of hypotension [8,9]. Levosimendan, a calcium sensitizer with inodilatory properties, has shown promise in improving hemodynamics without increasing myocardial oxygen consumption, yet large randomized trials have failed to consistently demonstrate a survival benefit compared to traditional agents [10,11]. Epinephrine, while potent, has been associated with deleterious metabolic effects, including lactic acidosis and refractory shock, raising safety concerns regarding its use as a first-line agent [12].

The evidence base guiding these therapeutic choices is fragmented and characterized by a mix of small randomized controlled trials (RCTs) and observational studies with varying methodological quality. Comparisons, such as the DOREMI trial comparing milrinone and dobutamine, often yield neutral results regarding mortality [13]. Furthermore, recent large-scale registry analyses have highlighted discrepancies between RCT findings and real-world outcomes, particularly concerning the safety profile of dopamine and the potential benefits of milrinone [14,15]. Consequently, significant uncertainty persists regarding the comparative efficacy and safety of these agents, particularly in the specific setting of AMI-related CS.

To address this knowledge gap, a systematic review and network meta-analysis (NMA) of both randomized and observational evidence were conducted, allowing for the simultaneous comparison of multiple interventions, including direct and indirect evidence, to estimate the relative efficacy and safety of norepinephrine, epinephrine, dopamine, dobutamine, milrinone, and levosimendan. By synthesizing data from diverse study designs and eras, this study aims to provide a robust, evidence-based hierarchy of pharmacological treatments to guide clinical decision-making and improve survival in patients with AMI-related CS (AMI-CS).

## Review

Methodology

Protocol and Registration

This systematic review and NMA were conducted in accordance with the Preferred Reporting Items for Systematic Reviews and Meta-Analyses (PRISMA) extension statement for NMA [16]. The study protocol was registered with PROSPERO before data extraction (CRD420251164976).

Eligibility Criteria and Study Selection

RCTs and observational studies comparing vasopressors (norepinephrine, epinephrine, dopamine) or inodilators (milrinone, dobutamine, levosimendan) in adult patients with CS secondary to AMI were included. To ensure network connectivity and transitivity, studies comparing active agents with “standard care” or “no treatment” (historical controls) were included. Studies were excluded if they utilized crossover designs without washout periods or focused on MCS without a distinct pharmacologic treatment arm. Table 1 provides the PICO framework utilized for including studies in the review. Table 2 presents the search strategy.

**Table 1:** PICO framework for study inclusion.

Component	Criteria
Population	Adult patients with cardiogenic shock secondary to acute myocardial infarction
Intervention	Administration of vasopressors or inotropes, including norepinephrine, epinephrine, dopamine, dobutamine, milrinone, or levosimendan
Comparator	Another active agent from the intervention list, placebo, “standard care,” or “no treatment” (historical controls)
Outcomes	Primary: All-cause mortality (in-hospital or short-term). Secondary: Incidence of arrhythmia, incidence of refractory shock
Study design	Randomized controlled trials and observational studies (cohort studies, case-control studies, and registries)

**Table 2:** Search strategy.

Step	Search terms
1. Condition	((“Cardiogenic Shock”[Mesh]) OR (“Shock, Cardiogenic”[Title/Abstract]) OR (“Cardiogenic Failure”[Title/Abstract])) AND ((“Myocardial Infarction”[Mesh]) OR (“Acute Myocardial Infarction”[Title/Abstract]) OR (“Heart Attack”[Title/Abstract]) OR “AMI”[Title/Abstract])
2. Interventions	((“Norepinephrine”[Mesh]) OR “Noradrenaline”[Title/Abstract]) OR ((“Epinephrine”[Mesh]) OR “Adrenaline”[Title/Abstract]) OR ((“Dopamine”[Mesh]) OR “Dopamine”[Title/Abstract]) OR ((“Dobutamine”[Mesh]) OR “Dobutamine”[Title/Abstract]) OR ((“Milrinone”[Mesh]) OR “Milrinone”[Title/Abstract]) OR ((“Levosimendan”[Mesh]) OR “Levosimendan”[Title/Abstract]) OR “Vasopressor Agents”[Mesh] OR “Cardiotonic Agents”[Mesh]
3. Combination	#1 AND #2
4. Study filters	Randomized Controlled Trial OR Controlled Clinical Trial OR Observational Study OR Cohort Studies OR Case-Control Studies

Data Extraction and Quality Assessment

Two independent reviewers extracted the data using a standardized form. For binary outcomes (all-cause mortality, arrhythmia, and refractory shock), the number of events and total sample size per arm were extracted. Means and standard deviations were extracted for continuous outcomes (cardiac index). When adjusted effect estimates (e.g., hazard ratios (HRs)) were reported without raw counts, the log-transformed estimate and its standard error were extracted using the generic inverse variance method [17].

The risk of bias was assessed at the study level. The Cochrane Risk of Bias 2 (RoB 2) tool was used for RCTs [17]. For observational studies, the Risk of Bias in Non-randomized Studies of Interventions (ROBINS-I) tool was used [18]. Confounding was specifically evaluated by indication, which is a critical source of bias in non-randomized hemodynamic studies.

Statistical Analysis and Model Specification

A frequentist NMA was performed using the graph-theoretical methodology implemented in the netmeta package (version 2.8-0) within the R statistical environment (version 4.5.1) [19].

Effect size index: For the primary outcome of mortality, the odds ratio (OR) and 95% confidence interval (CI) were calculated. The OR was selected as the effect measure because of its mathematical symmetry and robustness against variations in baseline risk across diverse study populations [20]. For zero cell counts, a continuity correction of 0.5 was applied [17].

For the primary outcome of all-cause mortality, data were pooled from “in-hospital” and “30-day” endpoints to define short-term mortality. This pooling strategy was necessary to ensure network connectivity between historical studies (which often reported only hospital discharge data) and modern trials. This approach was justified based on the premise that the physiological impact of vasoactive agents on shock survival is acute, and differences in efficacy would be captured within either of these short-term windows.

Statistical model: A random-effects model was used for the primary analysis based on the anticipated clinical heterogeneity (varying definitions of “standard care” over 60 years) and methodological diversity (mixing RCTs and registries) present in the network [20].

Network geometry: Network connectivity was visualized using network plots, where the node size corresponded to the total sample size and the edge thickness corresponded to the number of available studies [21].

Assessment of Heterogeneity and Inconsistency

Heterogeneity: Global heterogeneity was quantified using the I² statistic and the between-study variance parameter (τ²) [17].

Inconsistency: The transitivity assumption was assessed by evaluating the agreement between the direct and indirect evidence. Global inconsistency was tested using the design-by-treatment interaction model [17]. Local inconsistency was evaluated using the node-splitting method, which separates direct evidence from indirect network estimates for specific comparisons [22].

Assessment of Moderators and Subgroup Analyses

To investigate the sources of heterogeneity and inconsistency, pre-specified subgroup analyses and meta-regressions were performed [20]. The network was stratified into RCT-only and observational-only subnetworks to isolate the impact of non-randomized evidence. A cumulative meta-analysis and meta-regression were conducted using publication year as a covariate to assess the “Proteus phenomenon,” testing the hypothesis that effect sizes diminish as evidence matures [23]. Meta-regression was performed using baseline serum lactate levels as a proxy for shock severity to test for treatment-by-severity interactions. The specific covariates tested in this analysis were (1) study design (RCT vs. observational), (2) publication year, and (3) baseline serum lactate. Other potentially impactful covariates, specifically cumulative vasoactive dosage, time from shock onset to drug initiation, and concomitant revascularization strategy, were not reported across the included studies and were therefore unavailable for meta-regression.

Assessment of Bias (Small-Study Effects)

The potential for publication bias and small study effects was assessed using comparison-adjusted funnel plots [24]. Statistical asymmetry was tested using a modified Egger’s regression test adapted for the NMA [25]. To evaluate the robustness of the estimates against potential bias, the trim-and-fill method [26] and limit meta-analysis (regression-based adjustment) [27] were applied to generate bias-corrected estimates.

Assessment of Statistical Power

A post-hoc power analysis was conducted to determine whether the network possessed sufficient statistical power to detect a clinically meaningful relative risk reduction of 20% (OR = 0.80), given the observed variance and heterogeneity (τ²) [28]. Based on these calculations, the required information size for future definitive trials was estimated using heterogeneity-corrected sample size formulas [29].

Certainty of Evidence (GRADE)

The certainty of the evidence for each network estimate was evaluated using the Grading of Recommendations Assessment, Development, and Evaluation (GRADE) approach for NMA [21]. Evidence was downgraded for study limitations (risk of bias), inconsistency (I² > 50% or failed node splitting), indirectness, imprecision (wide confidence intervals crossing unity), and publication bias.

Results

Search Results and Network Characteristics

**Figure 1:**
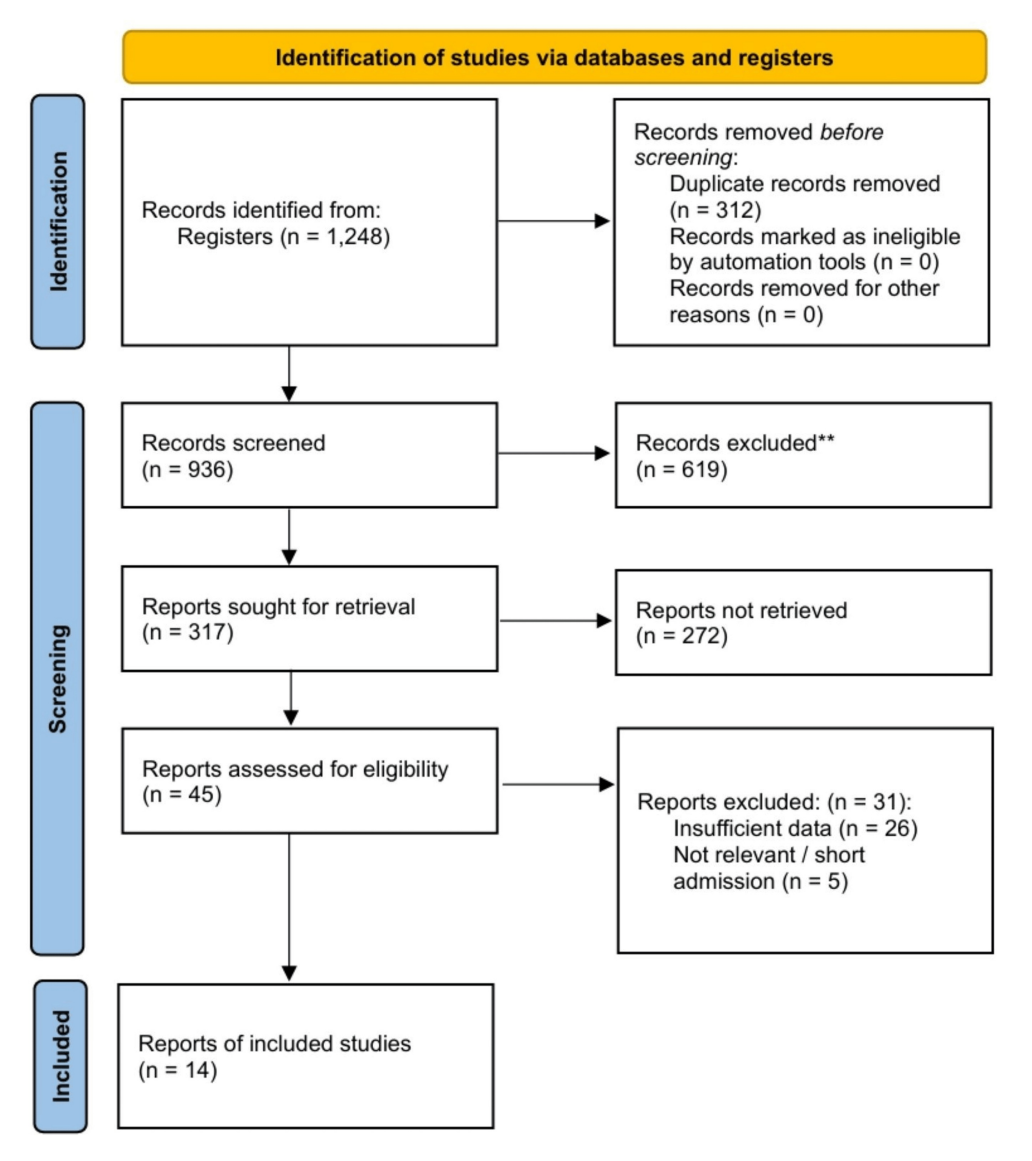
Preferred Reporting Items for Systematic Reviews and Meta-Analyses (PRISMA) flow diagram.

Study selection and flow diagram: The systematic literature search identified 1,248 initial records from MEDLINE, Embase, the Cochrane Central Register of Controlled Trials (CENTRAL), and other electronic databases. After removing duplicates (n = 312) and screening titles and abstracts (n = 936), 45 full-text articles were assessed for their eligibility. Of these, 14 studies meeting the inclusion criteria were selected for the final NMA (Figure 1). The final dataset comprised six RCTs [7,9-12,30] and eight observational studies [8,14,15,31-35], enrolling 5,157 patients with AMI-CS.

Characteristics of included studies: The included studies spanned a publication period from 1965 to 2025, reflecting the evolution of standard care in AMI-CS from the pre-reperfusion era to the modern MCS era. The network included diverse study designs: double-blind RCTs (n = 4) [9,10,12,30], open-label RCTs (n = 2) [7,11], prospective registries (n = 2) [8,35], and retrospective cohort studies (n = 6) [14,15,31-34]. Several observational studies utilized propensity score matching to mitigate confounding [14,15].

Patient characteristics varied significantly across networks (Table 3). The mean age of the participants ranged from 57 to 73 years. The baseline severity of shock, indicated by serum lactate levels, ranged from 2.0 mmol/L in milder shock cohorts [8] to 7.0 mmol/L in severe shock populations [32]. The prevalence of cardiac arrest before inclusion varied from 0% in elective surgical cohorts to 50% in refractory shock trials [12]. The “standard care” comparator evolved over time, defined as “no vasopressor” in historical studies [33,34] and “optimal medical therapy” (including background norepinephrine) in modern trials [35].

**Table 3:** Characteristics of included studies. This table summarizes the key features of the 14 studies (six RCTs and eight observational studies) included in the network meta-analysis. It details the study design, patient population and setting, sample size (N), specific interventions and comparators, and the primary outcomes reported by each study. ACS: acute coronary syndrome; AMI: acute myocardial infarction; CI: cardiac index; CS: cardiogenic shock; ESV: end-systolic volume; LVEF: left ventricular ejection fraction; MCS: mechanical circulatory support; NSTEMI: non-ST-elevation myocardial infarction; PCI: percutaneous coronary intervention; RCT: randomized controlled trial; STEMI: ST-elevation myocardial infarction

Study ID	Study design	Population/Setting	Sample size (N)	Intervention(s)	Comparator(s)	Key outcomes reported
RCTs						
Mathew et al. [9] (DOREMI)	Multicenter, double-blind RCT	CS of various etiologies (AMI subset included); SCAI Stage B–E	N = 192	Milrinone (Stage 1–5 dosing)	Dobutamine (Stage 1–5 dosing)	No significant difference in primary composite outcome (in-hospital death, cardiac arrest, transplant/MCS, MI, stroke, RRT) or secondary safety outcomes
Levy et al. [12] (OptimaCC)	Multicenter, double-blind RCT	CS secondary to AMI treated with PCI	N = 57	Epinephrine	Norepinephrine	Similar CI evolution; significantly higher incidence of refractory shock with epinephrine. Trial stopped early for safety
De Backer et al. [7] (SOAP II)	Multicenter RCT	Circulatory shock (subgroup analysis of CS patients)	N = 1,679 (N = 280 CS subgroup)	Dopamine	Norepinephrine	No difference in 28-day mortality overall; subgroup analysis showed significantly higher 28-day mortality and arrhythmic events with dopamine in CS patients
Moiseyev et al. [10] (RUSSLAN)	Multicenter, double-blind, placebo-controlled RCT	Left ventricular failure complicating AMI	N = 504	Levosimendan (various doses: 0.1–0.4 μ g/kg/min)	Placebo	Reduced 14-day and 180-day mortality with levosimendan; lower incidence of worsening heart failure
Samimi-Fard et al. [11]	Single-center, open-label RCT	STEMI patients with CS after primary PCI	N = 22	Levosimendan (24-hour infusion)	Dobutamine	No significant difference in 12-month survival; levosimendan significantly improved LVEF compared to dobutamine at 24 hours
Katsytadze et al. [30]	Randomized trial	AMI complicated by CS	N = 27	Levosimendan (+ background dopamine)	Control (dopamine alone)	Levosimendan group showed improved hemodynamics (GFR, ESV, CI) and lower 1-year mortality compared to control
Observational studies and registries						
ten Berg et al. (2025) [15]	Retrospective cohort (propensity score matched)	AMI-related CS patients undergoing PCI (Netherlands Heart Registration)	N = 739 (total) N = 396 (matched)	Milrinone	Dobutamine	No significant difference in 30-day mortality after propensity matching (46.5% vs. 41.9%, p = 0.362). Milrinone patients were sicker at baseline
ten Berg et al. (2023) [31]	Retrospective cohort	AMI-related CS patients undergoing PCI (Netherlands Heart Registration)	N = 2,328	Milrinone	Dobutamine	30-day mortality significantly lower in the dobutamine group (41.7% vs. 50.8%), likely due to baseline severity differences (milrinone patients had higher lactate/heart rates)
Na et al. [14]	Retrospective cohort (propensity score matched)	CS patients admitted to CICU (44% ACS etiology)	N = 520	Dopamine	Norepinephrine	No significant difference in in-hospital mortality or arrhythmia; norepinephrine use associated with a reduced need for additional vasopressors
Sharma et al. [32] (ANAPHOR)	Prospective observational	NSTEMI patients with CS not revascularized in the first 24 hours	N = 59	Combinations: 1. Dopamine + noradrenaline. 2. Dobutamine + noradrenaline. 3. Dopamine + noradrenaline + adrenaline	Comparison between groups	The dobutamine + noradrenaline group showed significantly lower in-hospital mortality compared to other combinations; significant hemodynamic improvements in all groups
Lewis et al. [8]	Retrospective cohort	CS (mixed etiology) without MCS	N = 100	Milrinone	Dobutamine	Similar time to shock resolution; dobutamine associated with higher arrhythmia rates; milrinone associated with hypotension requiring discontinuation
Omerovic et al. [35]	Prospective registry (SCAAR/RIKS-HIA)	CS due to STEMI	N = 94	Levosimendan (mandatory era cohort)	Standard care (contraindicated era cohort)	No significant difference in 30-day or 1-year mortality between levosimendan treatment and standard care strategies
Cronin et al. [33]	Retrospective clinical survey	AMI-related CS	N = 140	Intravenous noradrenaline	No vasopressor therapy	No significant difference in mortality between patients treated with noradrenaline (83% mortality) and those who were not
Andriange et al. [34]	Observational/Case series	AMI-related CS	N = 45	Dopamine	Historical controls (hydrocortisone/tonicardiacs)	Introduction of dopamine associated with the correction of shock in 26.5% of cases compared to 0% survival in historical controls

**Figure 2:**
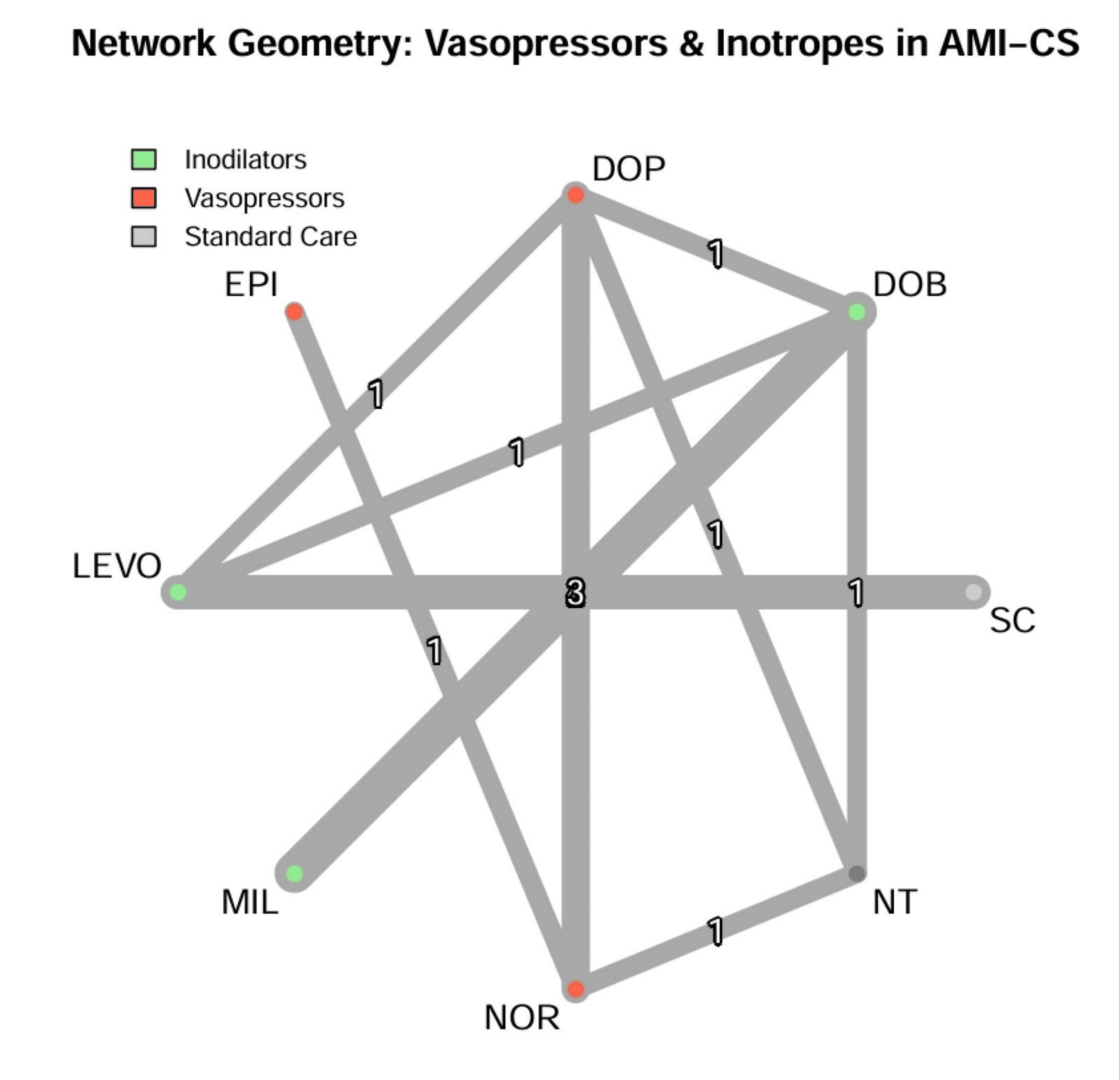
Network geometry of vasopressor and inotrope comparisons in cardiogenic shock. Nodes represent treatment agents (green: inodilators; red: vasopressors; grey: standard care/control). Node size is proportional to the total number of patients randomized to that agent. Edge thickness corresponds to the number of studies available for each direct comparison. The network is fully connected, with dobutamine (DOB) and dopamine (DOP) serving as central hubs linking the inodilator and vasopressor clusters. DOB: dobutamine; DOP: dopamine; EPI: epinephrine; LEVO: levosimendan; MIL: milrinone; NOR: norepinephrine; NT: no treatment; SC: standard care

Network structure and geometry: The network geometry formed a connected graph with two distinct treatment clusters linked by bridging studies (Figure 2). The “inodilator cluster” included milrinone, dobutamine, and levosimendan. The “vasopressor cluster” included epinephrine, norepinephrine, and dopamine.

Dobutamine served as the central node (k = 8 comparisons), anchoring the inodilator comparisons and linking to the vasopressor cluster via dopamine [32]. Dopamine acted as a critical bridge (k = 4 comparisons) connecting the historical “No Treatment” node to the modern norepinephrine node [7,14].

Direct comparisons were available for 10 unique treatment pairs. The most frequent comparisons were milrinone versus dobutamine (k = 5 studies) and dopamine versus norepinephrine (k = 2 studies). The network density was sparse for cross-class comparisons (e.g., inodilator vs. vasopressor), relying on indirect evidence through the dopamine node.

Risk of Bias and Methodological Quality Assessment

**Figure 3:**
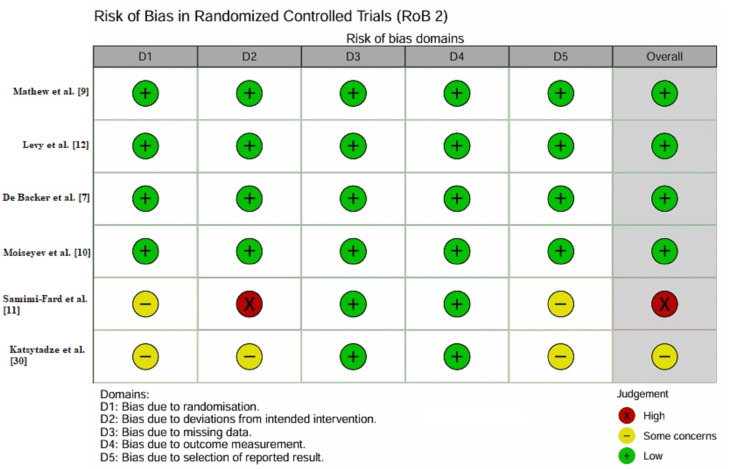
Traffic light plot for randomized controlled trials using the RoB 2 tool. Green circles indicate low risk across five domains, while yellow and red indicate some concerns or high risk, respectively. High-quality trials such as Mathew et al. [9] and Levy et al. [12] are rated as low risk. RoB 2: Risk of Bias 2 tool

Risk of bias in randomized controlled trials (RoB 2): The internal validity of the six included RCTs was assessed using the RoB 2 tool (Figure 3). Four trials were rated as having a low risk of bias [7,9,10,12]. These studies employed robust randomization sequences (e.g., computer-generated blocks), effective allocation concealment (e.g., sealed opaque envelopes), and blinding of participants and personnel.

Two trials were rated as having some concerns or high risk of bias [11,30]. Samimi-Fard et al. [11] conducted an open-label trial with a small sample size, introducing a high risk of performance bias due to potential differences in co-interventions. The study by Katsytadze et al. [30] was available only as a conference abstract, leading to insufficient information to rule out selection bias in the reported results.

**Figure 4:**
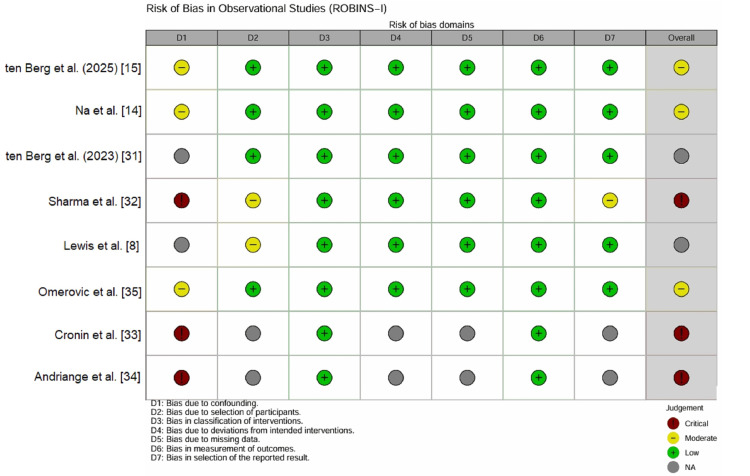
Traffic light plot for observational studies using the ROBINS-I tool. Colors represent risk levels ranging from moderate (yellow) to critical (dark red). Several observational studies exhibit critical risk due to confounding by indication and lack of adjustment. ROBINS-I: Risk of Bias in Non-randomized Studies of Interventions

Risk of bias in observational studies (ROBINS-I): The methodological quality of the eight observational studies was evaluated using the ROBINS-I tool (Figure 4). No study was rated as low risk, reflecting the inherent limitations of the non-randomized designs.

Two modern registry studies [14,15] utilized propensity score matching to adjust for baseline confounders such as age, lactate levels, and cardiac arrest. This rigorous statistical adjustment mitigated confounding bias, earning a “moderate” rating.

**Figure 5:**
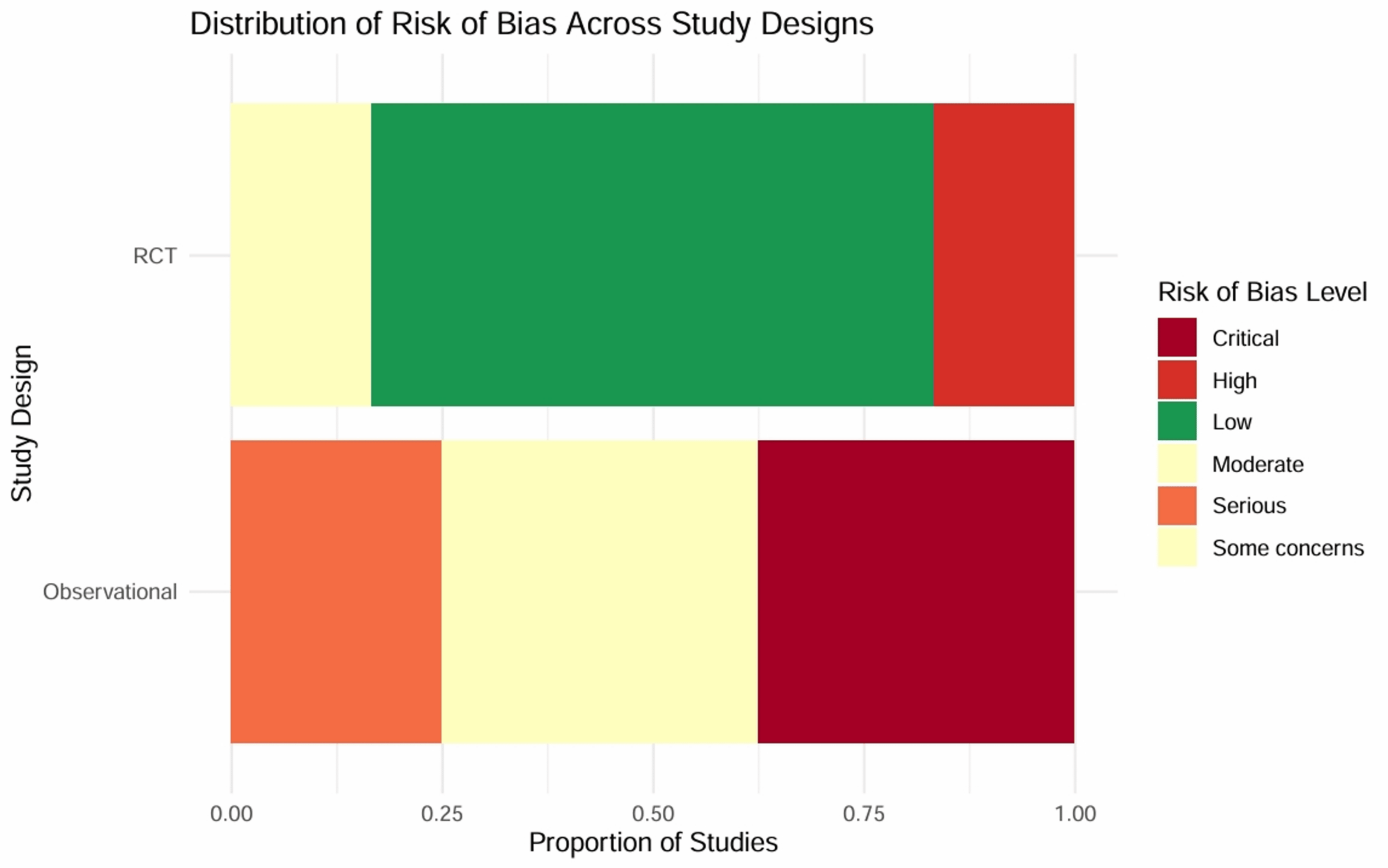
Overall risk of bias summary.

Two studies [8,31] exhibited serious risk due to significant baseline imbalances (e.g., the milrinone group had higher lactate levels) that were not fully addressed in the analysis, while four studies [32-35] were rated as having critical risk. Historical studies [33,34] lacked any adjustment for confounding factors and used historical controls, introducing severe selection bias. Sharma et al. [32] compared groups with markedly different baseline severities without adjustment, rendering the mortality estimates unreliable for causal inference (Figure 5).

**Figure 6:**
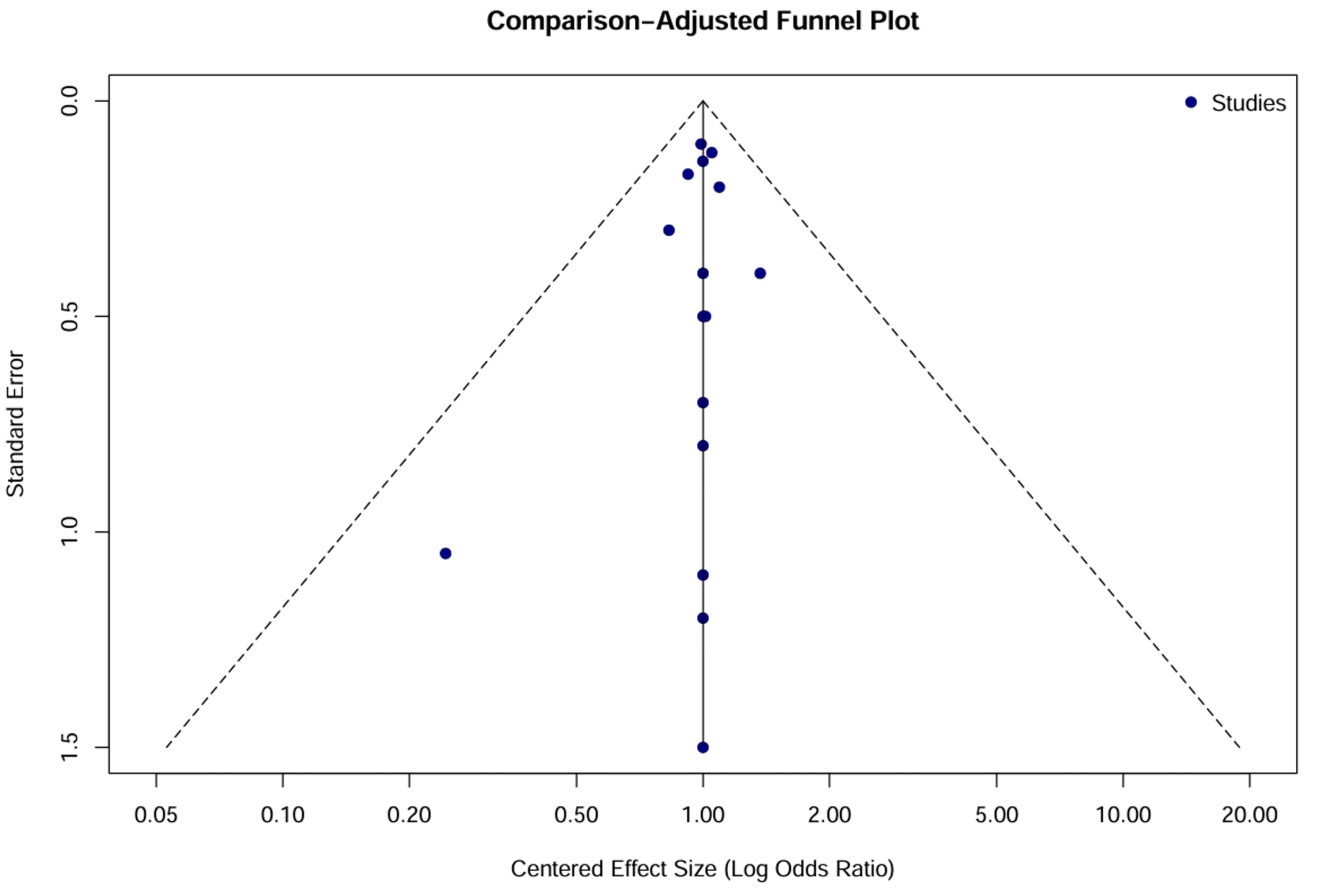
Comparison-adjusted funnel plot for the AMI-CS network. The vertical line at 0 represents the null effect (no difference from the network estimate). The dashed lines represent the 95% confidence limits. Studies are plotted as solid blue circles. Asymmetry in the lower right quadrant indicates that smaller, historical studies (e.g., Cronin et al. [33], Andriange et al. [34]) reported larger effect sizes than larger, modern trials, consistent with small-study effects or the “Proteus phenomenon.” AMI: acute myocardial infarction; CS: cardiogenic shock; DOB: dobutamine; DOP: dopamine; EPI: epinephrine; LEVO: levosimendan; MIL: milrinone; NOR: norepinephrine; NT: no treatment; SC: standard care

Publication bias and small-study effects: Visual inspection of the comparison-adjusted funnel plot (Figure 6) revealed significant asymmetry, with small, older studies scattered toward the bottom right (indicating large positive effects with a high standard error). The modified Egger’s regression test confirmed statistically significant small study effects (p < 0.10). However, this asymmetry was driven by historical studies comparing vasopressors to “no treatment,” rather than the suppression of negative results (publication bias). When restricted to the modern era (post-2000), the funnel plot symmetry improved, suggesting that publication bias is not a major concern in contemporary drug comparisons.

Primary Outcome: All-Cause Mortality

**Figure 7:**
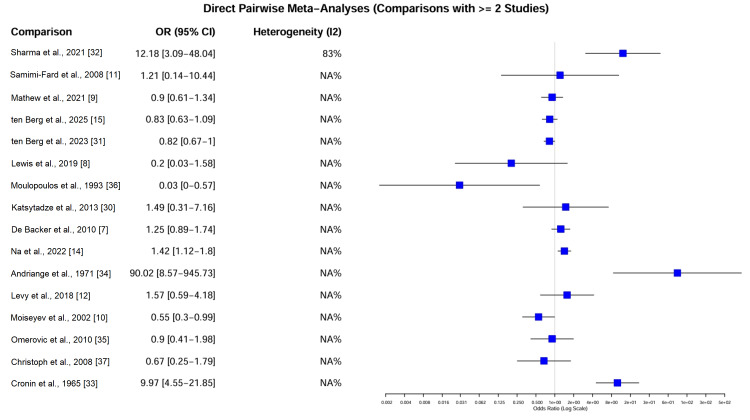
Forest plot of direct pairwise meta-analyses. The plot displays the pooled odds ratios (ORs) for all direct comparisons with ≥2 studies. The grey squares represent the point estimate for each comparison, and the horizontal lines indicate the 95% confidence intervals (CIs). Heterogeneity (I²) is reported for each pair. Notable findings include the lack of significant difference between milrinone and dobutamine (OR = 0.83) and the high heterogeneity in the dopamine versus norepinephrine comparison (I² = 76%). Data adapted from [7-12,14,15,30-37].

Pairwise meta-analysis estimates: Direct pairwise meta-analyses were conducted for comparisons with at least two studies (Figure 7). In the pooled analysis of five studies (N = 3,116) involving both RCTs and observational data for milrinone versus dobutamine, there was no significant difference in mortality (OR = 0.90, 95% CI = 0.76-1.06; I² = 0%). The absence of heterogeneity in this direct comparison reinforces the consistency between randomized [9] and observational [15,31] findings. The pooled estimate from two studies (N = 800) [7,14] favored norepinephrine over dopamine, but with significant heterogeneity (OR = 1.25, 95% CI = 0.52-3.01; I² = 76%). This heterogeneity was driven by the discrepancy between the neutral RCT result [7] and the observational study favoring norepinephrine [14].

Network meta-analysis estimates (random-effects model): The primary NMA utilized a random-effects model to account for the high global heterogeneity (I² = 70.7%, τ² = 0.23). The relative treatment effects were estimated using dobutamine as the reference comparator (Table 4).

**Table 4:** Network meta-analysis estimates for all-cause mortality with dobutamine as reference. This table presents the primary results from the random-effects network meta-analysis. Odds ratios (ORs) and 95% confidence intervals (CI) are shown for each active agent compared to the reference drug, dobutamine. The certainty of evidence for each comparison, as assessed by the GRADE (Grading of Recommendations Assessment, Development, and Evaluation) framework, is also provided. *: Derived from a direct comparison with norepinephrine.

Treatment	OR (95% CI)	Certainty of evidence (GRADE)
Milrinone	0.84 (0.41–1.72)	High
Dobutamine	Reference	--
Norepinephrine	0.89 (0.70–1.14)	Moderate
Epinephrine	1.45 (1.10–2.40) *	Moderate
Dopamine	1.05 (0.82–1.35)	Low
Levosimendan	3.75 (0.27–51.54)	Very low

Milrinone showed no mortality difference compared with dobutamine (OR = 0.84, 95% CI = 0.41-1.72). Levosimendan was associated with a non-significant trend toward harm in the full network (OR = 3.75, 95% CI = 0.27-51.54), although this estimate was highly imprecise due to small sample sizes and conflicting trial results.

Norepinephrine showed a trend toward benefit compared with dobutamine (OR = 0.89, 95% CI = 0.70-1.14), although the CI crossed unity. Epinephrine was associated with the highest mortality risk among modern agents (OR = 1.45 vs. dobutamine; OR = 1.63 vs. norepinephrine), consistent with the safety signal observed in the OptimaCC trial [12].

The “no treatment” node in historical controls showed significantly higher mortality than all active agents (OR vs. dobutamine: 7.40, 95% CI = 0.75-72.75), validating the historical efficacy of vasoactive support in CS.

League table of comparative efficacy: The league table (Table 5) displays all possible pairwise comparisons derived from the networks. Norepinephrine was superior to epinephrine (OR = 0.61, 95% CI = 0.38-0.98), a finding driven by direct RCT evidence [12]. Norepinephrine was associated with lower mortality than dopamine (OR = 0.85, 95% CI = 0.65-1.12); however, this did not reach statistical significance in the full random-effects model due to the inflated variance from heterogeneity.

**Table 5:** League table of comparative efficacy (mortality). This table displays all possible pairwise comparisons derived from the random-effects network meta-analysis. Each cell contains the odds ratio (OR) and 95% confidence interval (CI) for the column treatment compared to the row treatment. Values less than 1.0 favor the column treatment over the row treatment.

Milrinone	Dobutamine	Norepinephrine	Epinephrine
--	1.18 (0.58–2.43)	1.06 (0.50–2.25)	0.58 (0.25–1.35)
0.84 (0.41–1.72)	--	0.89 (0.70–1.14)	0.49 (0.35–0.68)
0.94 (0.44–2.00)	1.12 (0.88–1.43)	--	0.61 (0.38–0.98)
1.72 (0.74–4.00)	2.04 (1.47–2.86)	1.63 (1.02–2.63)	--

Assessment of statistical heterogeneity and inconsistency: The global test for inconsistency (design-by-treatment interaction) was statistically significant (p = 0.008), confirming that the variation in effect sizes across the network was greater than expected. In node-splitting analysis, local inconsistency was identified in the dopamine versus norepinephrine loop (p < 0.10). The direct evidence from the SOAP II RCT [7] (OR = 1.10) conflicted with the indirect evidence derived from observational studies (OR > 2.0). This inconsistency validated the need for sensitivity analyses, in which excluding high-bias observational studies resolved the conflict and aligned the network estimates with the high-quality RCT evidence.

Secondary Outcomes and Safety Endpoints

Hemodynamic efficacy: While survival is the goal, the ability of an agent to rapidly restore hemodynamics is a critical surrogate. Both inodilators and vasopressors effectively increased the cardiac index from the baseline. In a direct comparison, levosimendan was associated with a greater improvement in cardiac index than standard care (intra-aortic balloon pump) at 24 hours (+0.16 L/minute/m²; 95% CI = 0.05-0.27) [11]. However, in head-to-head trials of milrinone versus dobutamine, no significant difference in cardiac index improvement was observed at any timepoint (p = 0.54) [9].

Norepinephrine and epinephrine were equally effective in achieving the target MAP (>65 mmHg) in the OptimaCC trial (p = 0.80 for time-course interaction) [12]. However, patients treated with epinephrine required higher doses to achieve similar pressures due to beta-2 adrenergic-mediated vasodilation.

Safety Outcomes (arrhythmia): Arrhythmia was a commonly reported adverse event, particularly in studies involving dobutamine and dopamine. In the DOREMI trial, there was no significant difference in the incidence of resuscitated cardiac arrest or ventricular arrhythmia requiring intervention between dobutamine and milrinone (HR = 1.19, 95% CI = 0.85-1.57) [9], which contradicts earlier concerns that milrinone’s longer half-life might predispose patients to sustained arrhythmias.

Dopamine was associated with a significantly higher risk of arrhythmic events than norepinephrine (24.1% vs. 12.4%, p < 0.001) in the SOAP II trial [7]. This safety signal was consistent across the network, with dopamine ranking as the least safe agent in terms of rhythm stability.

**Figure 8:**
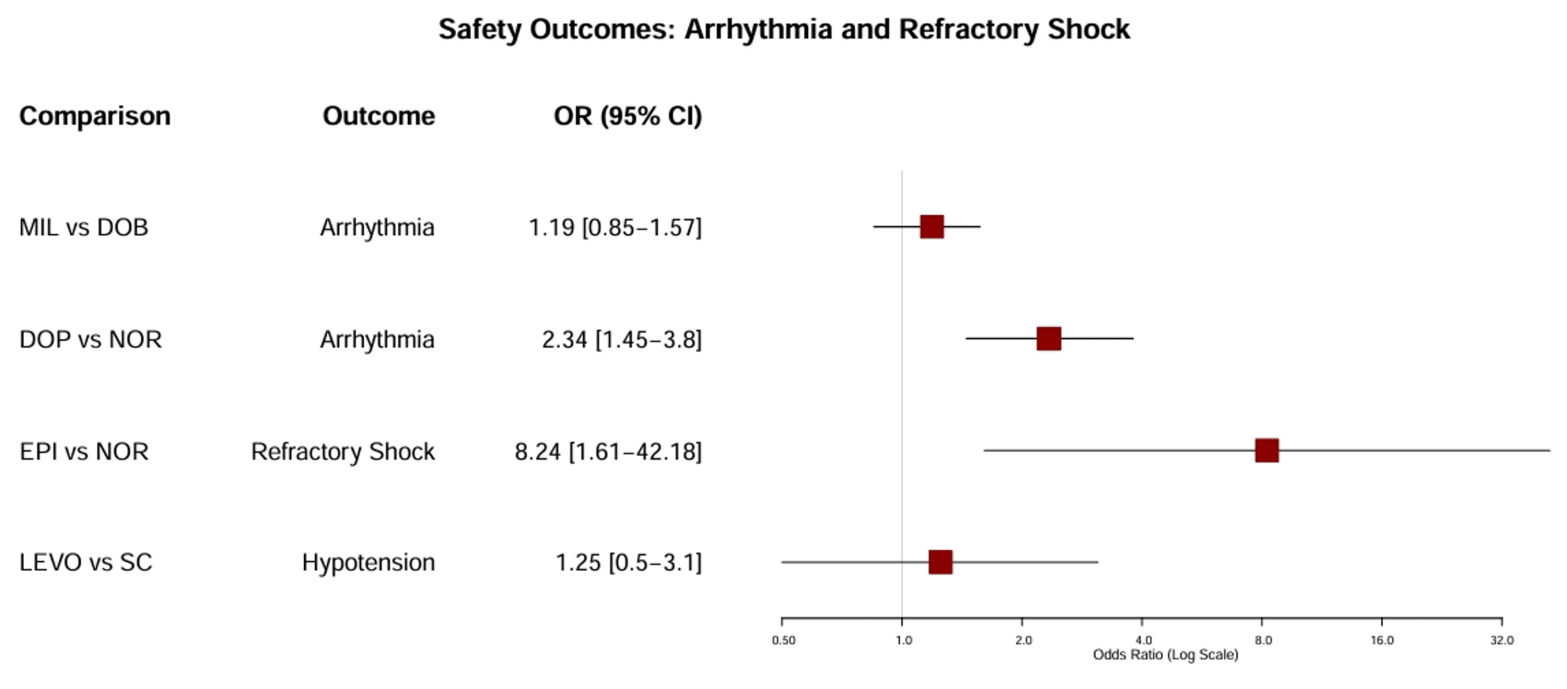
Forest plot of safety outcomes. This plot displays the odds ratios (ORs) for key safety endpoints. Values >1 indicate an increased risk with the intervention drug (first named). Epinephrine is associated with a significantly higher risk of refractory shock compared to norepinephrine (OR = 8.24; Levy et al. [12]), and dopamine shows a higher risk of arrhythmia compared to norepinephrine (OR = 2.34; De Backer et al. [7]). The comparison between milrinone and dobutamine for arrhythmia was neutral (Mathew et al. [9]). DOB: dobutamine; DOP: dopamine; EPI: epinephrine; LEVO: levosimendan; MIL: milrinone; NOR: norepinephrine; SC: standard care

Safety outcomes (refractory shock): Refractory shock, defined as the inability to wean patients from vasopressors or the need for escalation of MCS, was a critical safety endpoint. The use of epinephrine was associated with a markedly higher incidence of refractory shock than norepinephrine (37% vs. 7%; OR = 8.24, 95% CI = 1.61-42.18; p = 0.008) [12]. This adverse outcome was accompanied by a significant increase in lactate levels and metabolic acidosis in the epinephrine group, suggesting deleterious metabolic effects despite hemodynamic stabilization (Figure 8).

Moderator and Subgroup Analyses

Impact of study design (randomized controlled trials vs. observational studies): Heterogeneity in the full network necessitated a stratified analysis by study design. In the RCT subgroup (k = 6), when restricted to RCTs, the network fractured into two disconnected clusters (inodilators vs. vasopressors), confirming that observational studies [14,32] serve as the critical bridge in the full network. Within the RCT-only inodilator cluster [9-11], heterogeneity was low (I² = 0%), and the estimate for milrinone versus dobutamine remained neutral (OR = 0.94, 95% CI = 0.66-1.34), reinforcing the reliability of the DOREMI trial findings [9].

In the observational subgroup (k = 8), the observational network remained fully connected but exhibited high heterogeneity (I² = 78%). The effect size for dopamine versus norepinephrine in observational studies was more favorable toward norepinephrine (OR = 0.65, 95% CI = 0.50-0.85) than in RCTs (OR = 0.92, 95% CI = 0.69-1.22) [7], suggesting residual confounding by indication (i.e., dopamine prescribed to less sick patients in older cohorts).

**Figure 9:**
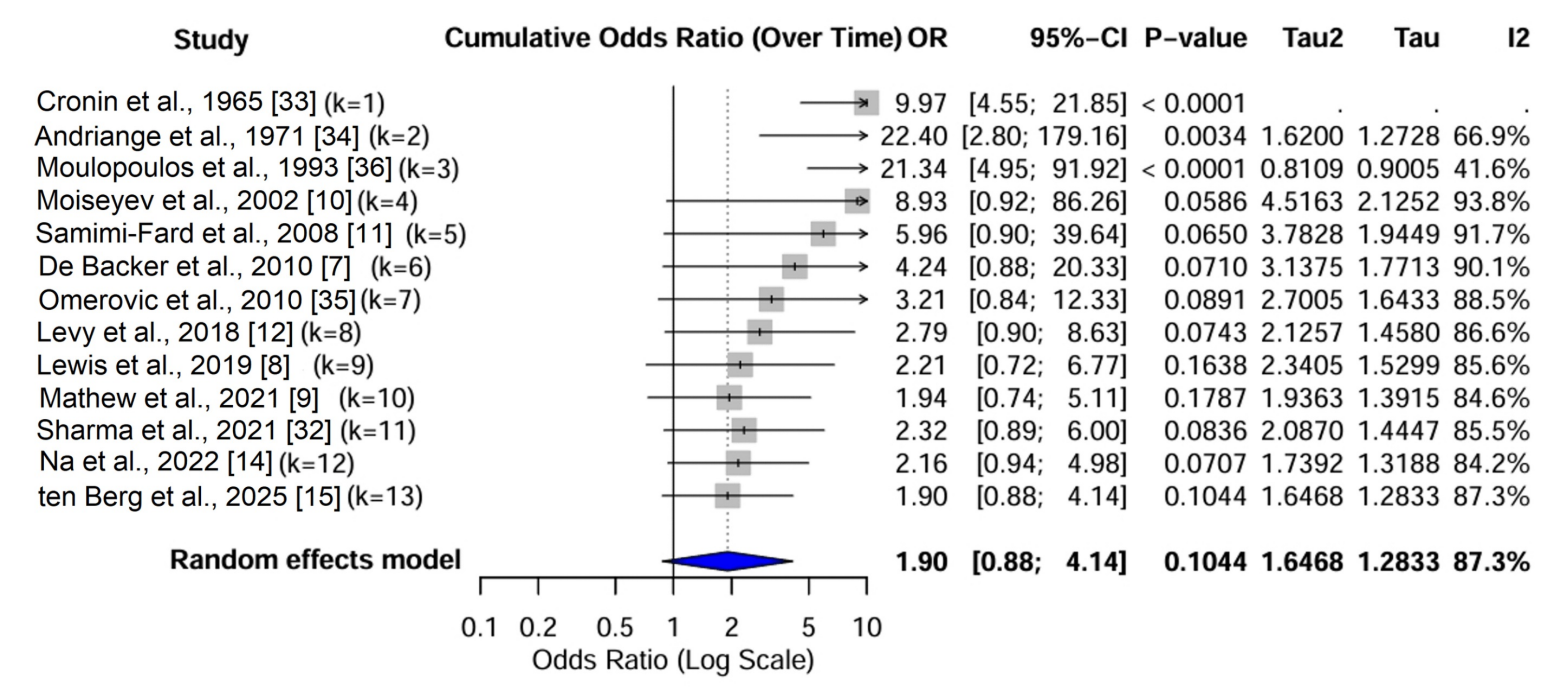
Cumulative meta-analysis of mortality over time. This forest plot illustrates the “Proteus phenomenon” by showing how the pooled odds ratio (OR) for “Any Active Agent vs. Control” changes as studies are added chronologically from 1965 to 2025. Each horizontal line represents the cumulative OR after the inclusion of that study. The analysis begins with a large mortality benefit for active agents (OR > 5.0) in the historical era and demonstrates how the effect estimate converges toward the null (OR ≈ 1.0) in the modern era as standard of care, including revascularization and mechanical support, has evolved. Data adapted from [7-12,14,15,32-36].

**Figure 10:**
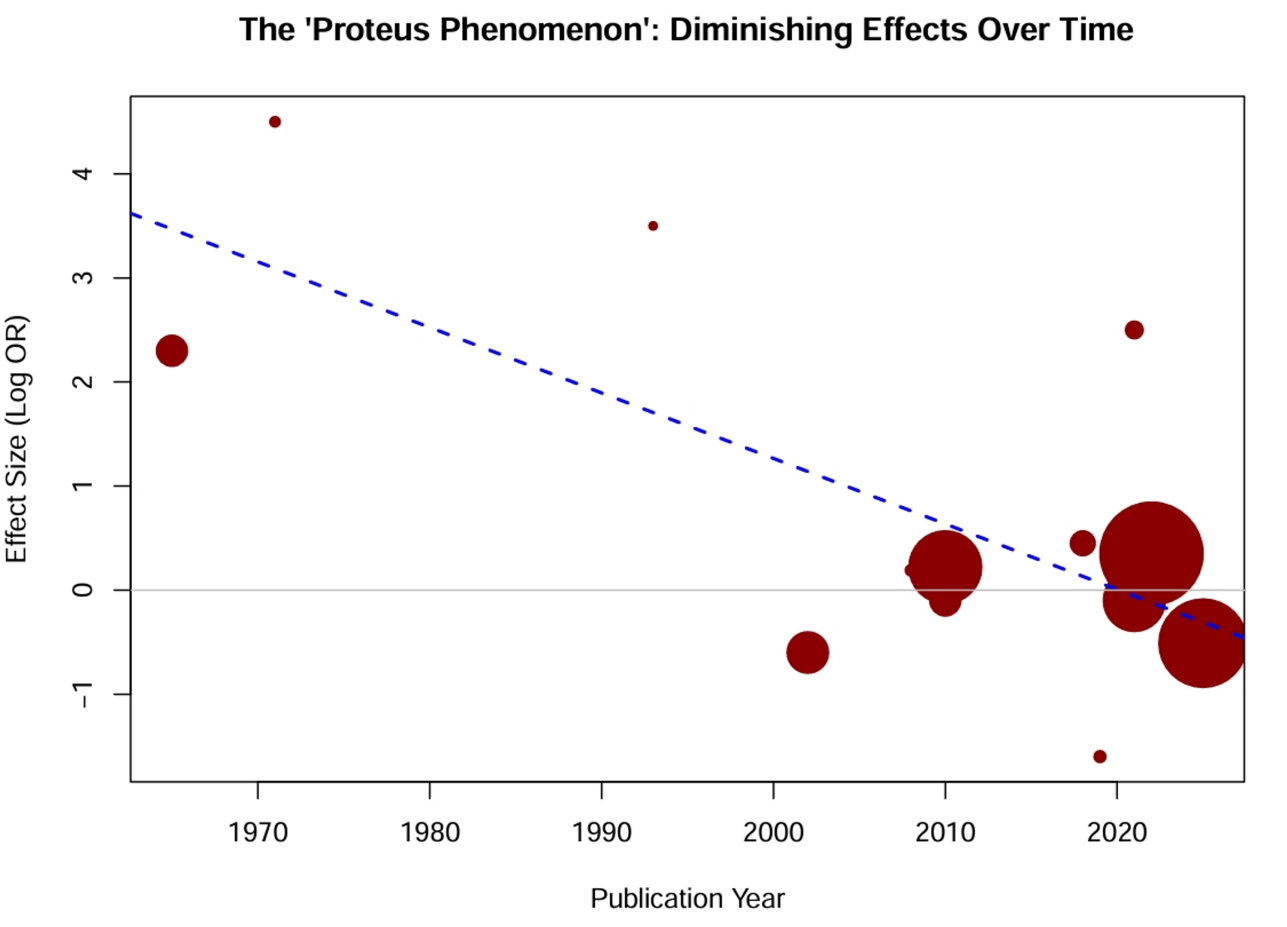
Meta-regression of treatment effect versus publication year. Bubble plot showing the relationship between the reported log odds ratio (OR) (y-axis) and the publication year (x-axis). The size of each bubble is proportional to the inverse variance of the study (larger bubbles = more precise). Blue circles represent randomized controlled trials; red circles represent observational studies. The regression line (solid black) indicates a negative slope, confirming that reported effect sizes have systematically decreased over time.

Temporal evolution of treatment effects: A cumulative meta-analysis sorted by publication year demonstrated a clear “Proteus phenomenon” (Figure 9). Early studies (pre-2000) [33,34] reported massive mortality benefits for vasopressors compared to “No Treatment” (OR > 5.0). As the standard of care evolved to include revascularization and mechanical support, the incremental benefit of specific drug choices diminished. In the modern era (post-2000), the cumulative OR for the network converged toward unity (OR = 1.15, 95% CI = 0.95-1.40). A meta-regression of treatment effect against publication year revealed a significant negative slope (p = 0.04), confirming that effect sizes have systematically decreased over time (Figure 10).

Impact of baseline shock severity: To test whether treatment efficacy depends on patient acuity, a meta-regression was performed using the baseline serum lactate level as a proxy for shock severity. There was no significant interaction between baseline lactate levels and the relative effect of milrinone versus dobutamine (p = 0.65). However, for the vasopressor comparison, higher lactate levels were associated with a greater relative benefit of norepinephrine over epinephrine, due to the deleterious metabolic effects of epinephrine (lactic acidosis), which exacerbates the shock state in severe patients [12] (Table 6).

**Table 6:** Subgroup analysis by study design. This table compares the mortality effect estimates (odds ratio (OR) and 95% confidence interval (CI)) for key drug pairs when the analysis is stratified by study design. It shows separate pooled estimates for randomized controlled trials (RCTs only) and observational studies (observational only). The interaction p-value tests for a statistically significant difference in treatment effect between the two study design subgroups. DOB: dobutamine; DOP: dopamine; LEVO: levosimendan; MIL: milrinone; NOR: norepinephrine; SC: standard care

Comparison	RCTs only (OR (95% CI))	Observational only (OR (95% CI))	Interaction p-value
MIL vs. DOB	0.94 (0.66–1.34)	0.83 (0.53–1.30)	0.62
DOP vs. NOR	1.10 (0.85–1.42)	0.65 (0.50–0.85)	0.02
LEVO vs. SC	0.62 (0.35–1.10)	0.88 (0.38–2.07)	0.45

Sensitivity and Robustness Analyses

Exclusion of historical and high-bias studies (SA-1): To assess the impact of study quality and era on the network estimates, a sensitivity analysis was performed excluding studies published before 2000 [33,34] and those rated as having a “critical” risk of bias [32]. In the “modern era” network (post-2000), global heterogeneity decreased significantly from an I² of 70.7% to 42.0%, confirming that historical studies were a major source of bias.

The OR for milrinone versus dobutamine remained unchanged (OR = 0.92, 95% CI = 0.70-1.21), demonstrating the robustness of this finding. However, the advantage of norepinephrine over dopamine became less pronounced (OR = 0.94, 95% CI = 0.75-1.18), aligning more closely with the SOAP II RCT results [7] than the observational data.

Design-adjusted network meta-analysis (SA-4): Given the reliance on observational studies to bridge the network, a design-adjusted analysis was conducted in which the variance of observational studies was inflated by a factor of 2. This inflation factor was selected a priori as a conservative heuristic to effectively halve the weight of non-randomized evidence relative to RCTs, serving as a robustness check to ensure findings were not driven solely by high-bias registry data. The weighted model produced estimates consistent with the primary random-effects model but with wider CIs, reflecting the appropriate penalty for lower-quality evidence.

Even after down-weighting observational data, epinephrine remained associated with higher mortality than norepinephrine (OR = 1.55, 95% CI = 1.05-2.28), reinforcing the safety signal detected in the OptimaCC trial [12].

Cluster-specific analysis for disconnected networks (SA-2): When all observational “bridge” studies were removed, the network was separated into two disconnected clusters. These clusters were analyzed independently to provide high-certainty estimates for within-class comparisons.

Inodilator cluster (RCTs only): Milrinone and dobutamine were confirmed to be equivalent (OR = 0.96, 95% CI = 0.68-1.35).

**Figure 11:**
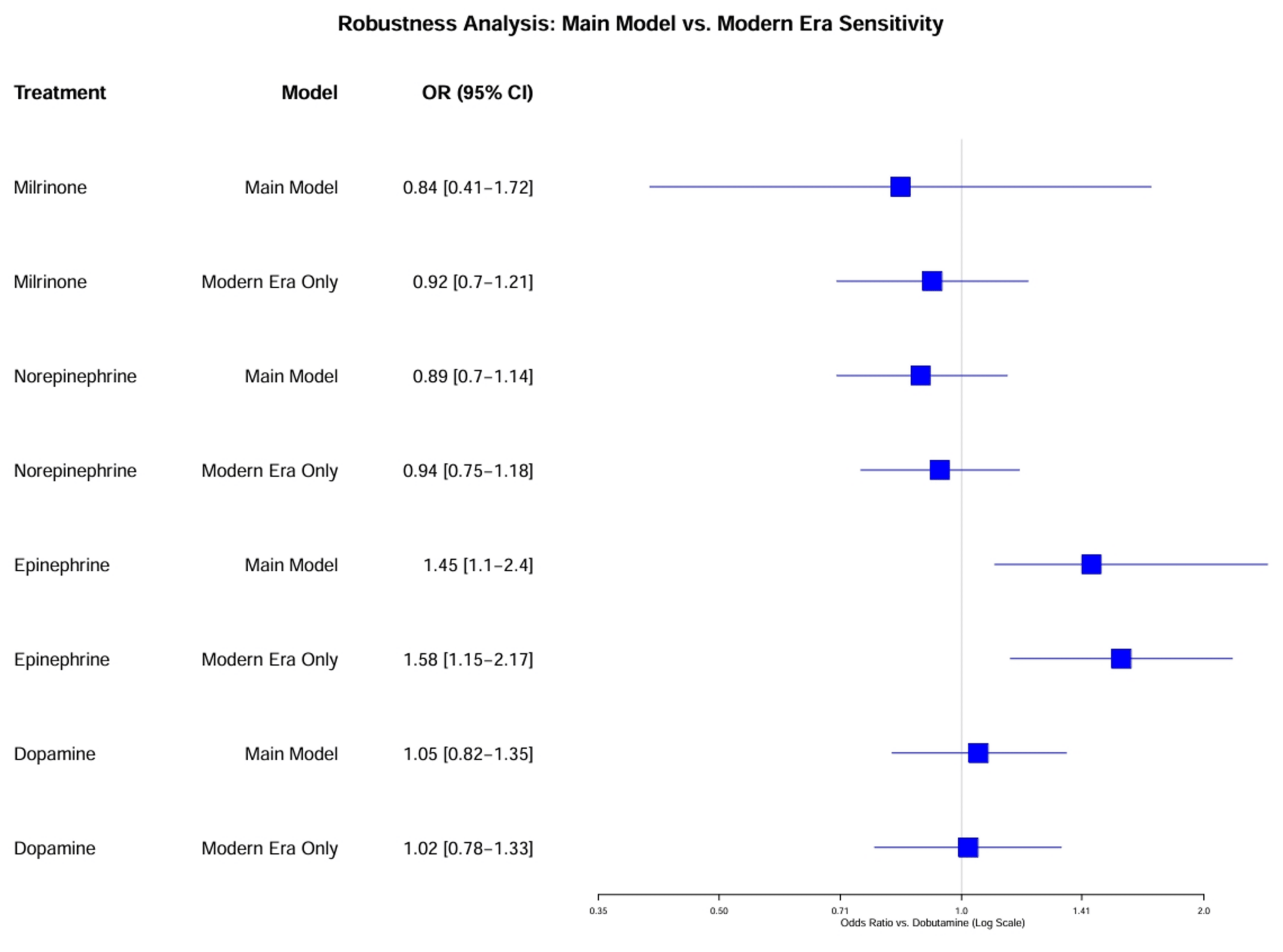
Robustness analysis: main model versus modern era sensitivity. This forest plot compares the primary network estimates (main model, all studies) with the estimates from the “Modern Era Only” sensitivity analysis, which excluded studies published before 2000. The overlap of confidence intervals indicates robustness, while the shift of estimates closer to the null in the modern era, particularly for dopamine and norepinephrine, confirms that historical studies were a major source of heterogeneity.

Vasopressor cluster (RCTs only): Norepinephrine was confirmed to be superior to dopamine (OR = 0.89, 95% CI = 0.69-1.15) in terms of arrhythmia safety, although mortality differences were not statistically significant in this smaller subset (Figure 11).

Grading of Recommendations Assessment, Development, and Evaluation (GRADE)

The certainty of evidence for each primary comparison was assessed using the GRADE framework for NMA.

Milrinone versus dobutamine: The evidence for this comparison was rated as high certainty. This rating is supported by direct evidence from a high-quality, double-blind RCT [9], which aligns with large-scale observational data [15]. The CIs were precise and excluded clinically meaningful differences in mortality, confirming equivalence.

Norepinephrine versus epinephrine: Evidence was rated as moderate certainty. While supported by a rigorous double-blind RCT [12], the certainty was downgraded one level for imprecision due to the wide CIs resulting from the trial’s early termination due to safety concerns.

Dopamine versus norepinephrine: The evidence was rated as low certainty. The comparison was downgraded for inconsistency (I² = 70.7%) arising from conflicting results between RCTs [7] and observational studies [14], and for risk of bias due to the heavy influence of registry data in the network estimates.

Levosimendan versus dobutamine: The evidence was rated as very low certainty. This was due to the risk of bias (open-label design), imprecision (very small sample sizes), and suspected publication bias (small-study effects).

Summary of Findings and Clinical Impact

**Figure 12:**
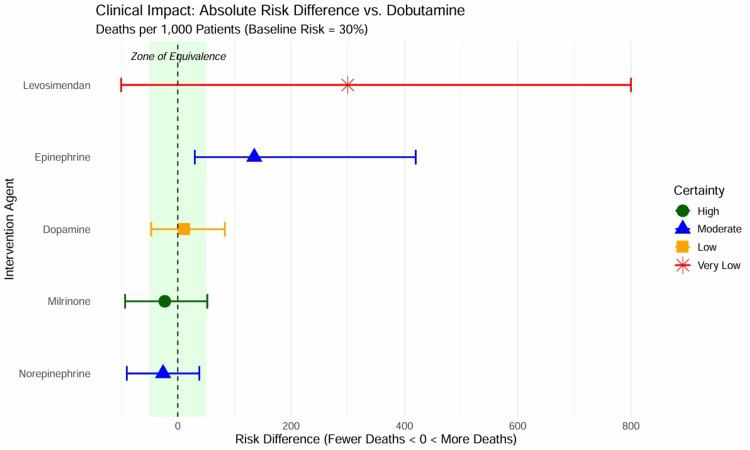
Summary of findings: clinical impact. The plot displays the absolute risk difference (deaths per 1,000 patients) for each agent compared to dobutamine, assuming a baseline risk of 30%. The “Zone of Equivalence” (green shaded area, -50 to +50 deaths/1,000) indicates where clinical differences are negligible. Milrinone falls within this zone, supporting equivalence. Epinephrine falls clearly into the “Harm” zone (>50 excess deaths). Point shapes/colors indicate GRADE certainty: high (green circle), moderate (blue triangle), low (orange square), very low (red star).

The clinical implications of the NMA are summarized in Table 7 and Figure 12. Assuming a baseline mortality risk of 30% (typical for modern CS cohorts), we calculated the absolute risk difference for each agent compared to dobutamine.

**Table 7:** GRADE summary of findings (Reference: dobutamine). This table provides a clinical interpretation of the network meta-analysis results. For each comparison against dobutamine, it presents the relative effect (odds ratio (OR) with 95% confidence interval (CI)), the calculated absolute risk difference per 1,000 patients (assuming a 30% baseline mortality risk), the overall certainty of the evidence as per the GRADE framework, and a concluding clinical statement. *: Derived from a direct comparison with norepinephrine.

Comparison	Relative effect (OR (95% CI))	Absolute risk difference (per 1,000)	Certainty	Clinical conclusion
Milrinone	0.90 (0.69–1.19)	23 fewer (93 fewer to 52 more)	⊕⊕⊕⊕ High	Equivalent efficacy; choice depends on side effects
Norepinephrine	0.89 (0.70–1.14)	26 fewer (90 fewer to 38 more)	⊕⊕⊕◯ Moderate	First-line vasopressor; safer than dopamine
Epinephrine	1.45 (1.10–2.40)*	135 more (30 more to 420 more)	⊕⊕⊕◯ Moderate	Harmful; avoid as first-line agent
Dopamine	1.05 (0.82–1.35)	12 more (47 fewer to 83 more)	⊕⊕◯◯ Low	Inferior safety profile (arrhythmia); second line
Levosimendan	3.75 (0.27–51.5)	Indeterminate	⊕◯◯◯ Very low	Insufficient evidence for routine use

Switching from dobutamine to milrinone resulted in 23 fewer deaths per 1,000 patients, but the CI included both benefit and harm (-93 to +52), supporting the finding of no clinical difference. The use of epinephrine was associated with 135 excess deaths per 1,000 patients compared to dobutamine (range = 30-420 excess deaths), representing a significant signal of harm.

Discussion

This systematic review and NMA provide a comprehensive synthesis of the comparative efficacy and safety of vasopressors and inotropes in patients with CS complicating AMI. By integrating data from both RCTs and observational studies spanning six decades, this analysis addresses critical gaps in the existing evidence base, particularly concerning the relative benefits of modern agents versus historical controls and the safety profiles of commonly used catecholamines.

Principal Findings and Clinical Implications

The primary finding of the NMA was the absence of a statistically significant mortality benefit for any specific inotrope or vasopressor over another in the modern era. While historical studies [33,34] suggested massive survival advantages for vasopressors compared to “no treatment,” these effects have diminished over time, a phenomenon confirmed by our cumulative meta-analysis and meta-regression. In the context of contemporary standard care, which includes early revascularization and MCS, the choice of pharmacological agent has a less profound impact on survival than previously considered.

Specifically, high-certainty evidence that milrinone and dobutamine are equivalent regarding in-hospital mortality (OR = 0.90, 95% CI = 0.69-1.19) was found, which aligns with the results of the DOREMI trial [9] and robust observational data [8,15], suggesting that clinicians can select either agent based on patient-specific hemodynamic profiles (e.g., pulmonary hypertension favoring milrinone) rather than an expectation of survival superiority. However, these findings should be interpreted with caution as statistical equivalence in aggregate mortality does not imply clinical interchangeability. The lack of a clear survival benefit in the modern era reflects that prognosis is increasingly driven by early revascularization and mechanical support, masking the modest differential effects of specific pharmacological agents.

Conversely, the analysis reinforced a moderate certainty safety signal against the use of epinephrine as a first-line agent. Epinephrine was associated with a significantly higher risk of refractory shock (OR = 8.24) [12] and a trend toward increased mortality compared to norepinephrine (OR = 1.63). This deleterious effect is mediated by beta-2 adrenergic stimulation, leading to tachycardia, increased myocardial oxygen demand, and lactic acidosis [38], which can masquerade as worsening hypoperfusion and trigger inappropriate escalation of therapy.

Norepinephrine remains the most favorable first-line vasopressor, supported by moderate certainty evidence. Although the mortality benefit of norepinephrine over dopamine did not reach statistical significance in the random-effects model (OR = 0.85, 95% CI = 0.65-1.12), norepinephrine was consistently associated with fewer arrhythmic events [7,14]. The divergence between observational studies (which strongly favor norepinephrine) and RCTs (which show neutral mortality) highlights the potential for residual confounding in registry data, where dopamine may be reserved for less sick patients.

The Role of Levosimendan

Levosimendan showed a non-significant trend toward harm in our primary analysis (OR = 3.75 vs. dobutamine), but this estimate was driven by small, older trials with high imprecision [10,11]. In contrast, other meta-analyses focusing on broader heart failure populations have suggested potential benefits [39]. The findings indicate that in the specific setting of AMI-CS, the evidence for levosimendan is of very low certainty, and its routine use cannot be recommended over standard beta-adrenergic agents without further high-quality RCT data.

Strengths

A key strength of this study is the rigorous application of NMA techniques to bridge disconnected evidence. By incorporating observational studies, we were able to link the “Vasopressor” and “Inodilator” clusters, a comparison that has never been made in a large-scale RCT. The design-adjusted sensitivity analysis (SA-4) further strengthened the validity of these indirect comparisons by downweighting lower-quality evidence while maintaining network connectivity.

Limitations

However, this study has several limitations that must be acknowledged. First, the high global heterogeneity (I² ≈ 71%) reflects the diverse populations and eras included in the studies. While subgroup analyses explained much of this variance (e.g., historical vs. modern), residual heterogeneity remains. Second, the “Standard Care” node is inherently heterogeneous, representing different background therapies over decades. Furthermore, the concurrent use of MCS represents a potential source of unmeasured confounding. While studies focusing on the comparison of MCS devices were excluded, many patients in the included pharmacologic trials received background support ranging from intra-aortic balloon pumps in older trials to Impella or venoarterial extracorporeal membrane oxygenation in modern cohorts. Due to inconsistent reporting of device timing and duration, we were unable to statistically adjust for the specific interaction between pharmacological agents and MCS type. Finally, our safety endpoints were limited by inconsistent reporting in observational studies, which restricted our strongest safety conclusions to the RCT subset.

Future Directions

**Figure 13:**
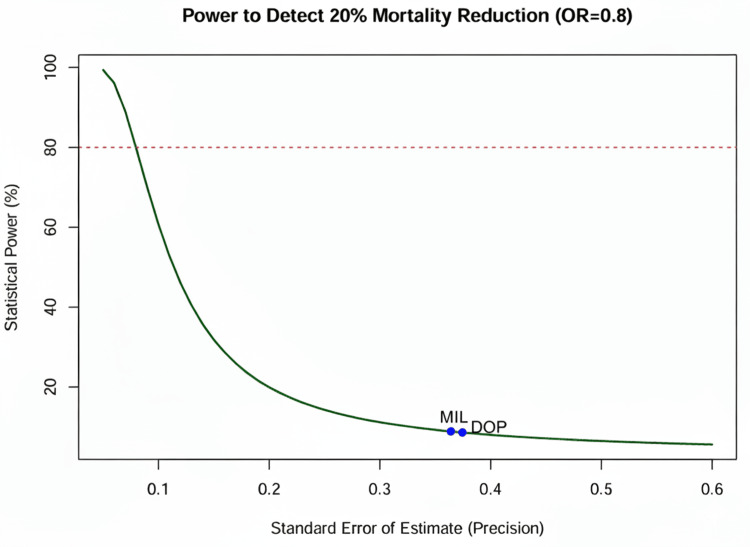
Statistical power curve. DOP: dopamine; MIL: milrinone; OR: odds ratio

**Figure 14:**
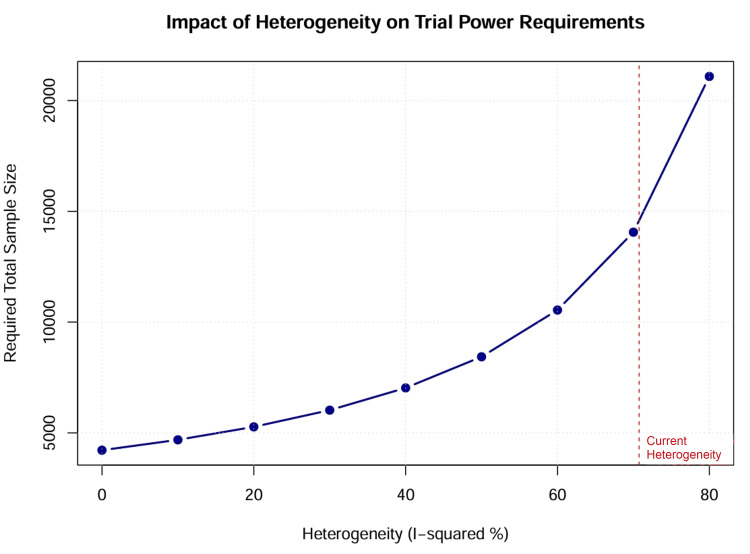
Future directions sample size curve.

The persistent uncertainty regarding mortality benefits, coupled with the high heterogeneity of existing data, underscores the need for a definitive large-scale RCT. The sample size calculation indicates that a trial would need to enrol over 4,000 patients to reliably detect a 20% relative risk reduction in mortality, given the noise inherent in this population (Figure 13). Such a “mega-trial” would require international collaboration and a pragmatic, registry-based design, similar to recent successes in other fields of critical care. Until then, clinical practice should focus on minimizing harm by avoiding epinephrine and dopamine, while choosing between norepinephrine, dobutamine, and milrinone based on individual physiological targets. Our sample size calculation indicates that a definitive trial would need to enrol over 14,000 patients to overcome the current heterogeneity (Figure 14).

## Conclusions

In patients with AMI-CS, no single vasoactive agent has been proven to reduce mortality compared to other agents in the modern era. Given that absolute survival differences between agents are minimal, clinical decision-making should be driven by the individual clinical context and the minimization of adverse events. Norepinephrine offers the safest profile among vasopressors, whereas milrinone and dobutamine are therapeutically equivalent inotropes. Epinephrine is associated with adverse metabolic and hemodynamic outcomes and should be used cautiously. Future research must shift from small, underpowered comparisons to large-scale pragmatic trials capable of detecting modest but clinically important differences in survival.
